# Fracture resistance and mode of failure of modified Polyether-ether-ketone versus lithium disilicate endocrowns

**DOI:** 10.1186/s12903-024-05232-3

**Published:** 2025-01-08

**Authors:** Mohamed G. A. Kharboush, Hesham I. Othman, Mohamed F. Aldamaty, Ahmed M. L. Alameldin

**Affiliations:** 1 Department of Fixed Prosthodontics, Faculty of Dental Medicine, Al-Azhar University, Cairo, 11651 Egypt; 2 Present Address: Department of Restorative and Aesthetic Dentistry, College of Dentistry, Almaaqal University, Basrah, Iraq

**Keywords:** Endocrown, Failure mode, Fracture resistance, Lithium disilicate, Polyether-ether-ketone

## Abstract

**Purpose:**

The current study aimed to compare modified Polyether-ether-ketone's fracture resistance and failure mode versus lithium disilicate glass–ceramic endocrowns.

**Materials and methods:**

A total of 16 butt-joint endocrown specimens on mandibular second molar teeth were fabricated and divided into two equivalent groups; Pressable modified Polyether-ether-ketone (PEEK) (BioHPP^®^) and Pressable lithium disilicate glass ceramic (IPS e.max^®^ Press). A computer-aided design/computer-aided manufacturing system was used to digitally create and milled wax patterns. Final restorations were cemented each to its corresponding prepared tooth. Thermomechanical cycling loading representing one year of clinical service was done in a chewing simulator. Fracture resistance was evaluated utilizing a universal testing machine. The failure mode was evaluated by inspecting fractured surfaces using a Stereomicroscope and further examined by a Scanning Electron Microscope (SEM) amongst both groups.

**Results:**

A statistically significant variation in fracture resistance was recorded with a mean load of (2762.96 ± 216.15 *N*) for modified PEEK and (2175.91 ± 267.72 *N*) for lithium disilicate glass–ceramic endocrowns.

**Conclusion:**

Modified PEEK endocrowns have higher fracture resistance than lithium disilicate glass–ceramic endocrowns. Moreover, the likeliness of catastrophic fracture in molars receiving endodontic treatment restored by modified PEEK is less than teeth restored with lithium disilicate glass ceramics.

## Introduction

Selecting the appropriate material and technique to restore endodontically treated teeth (ETT) with excessive coronal tooth structure loss is still a challenge [1]. For ETT teeth with extensive coronal damage, post and core restoration has been the standard procedure, accompanied by a full-coverage crown [2]. However, extensive removal from tooth structure during post-space preparation may further weaken the tooth [3].

Endocrown is a one-piece, post-free ceramic restoration that assembles the crown and the pulpal part in one component [4]. In comparison to the traditional post-and-core treatment, this modern alternative treatment modality offers a better chance for the preservation of tooth structure, more precisely the “Bio-Ring” of the peri-cervical dentin of the tooth [5]. Endocrown restorations don’t require large interocclusal space, with less chance of root fracture, and permit endodontic retreatment in case of failure. Additionally, there are fewer chairside steps [6]. It has been suggested that endocrowns be used for teeth with short clinical crowns and curved, short, or calcified root canals, which increase the risk of post and core restorations [7].

The advent of high-strength ceramic materials, computer-aided design computer-aided manufacturing (CAD-CAM), and the progress made in adhesive dentistry make lithium disilicate (LDS) reinforced etchable glass ceramics the preferred material in endocrown restorations [8], which provide excellent esthetics, high mechanical and flexural strength (440 MPa) adequate to withstand the occlusal load, high modulus of elasticity (95 GPA), and adequate bond strength to the tooth structure [9]. LDS can be manufactured from either pressable ingots or CAD-CAM machinable blocks [10]. The inherent brittleness and rigidity of dental ceramics have been a common cause of failure for restorations on ETT [11].

PEEK is a linear, semi-crystalline thermoplastic aromatic core connected by ether and ketone groups which provide superior chemical stability [12]. To enhance PEEK's mechanical characteristics, filler modifications were made to the PEEK. High-Performance Polymer (BioHPP^®^), a modified PEEK with 20% ceramic additives, is not allergic and possesses excellent stability, excellent biocompatibility, and great optimal polishability enabling the production of prosthetic dental restorations [13]. The grain size of the ceramic filler ranges from 0.3 to 0.5 μm. This minuscule grain size allows the generation of uniformity of structure with improved strength and abrasion properties. The increased interest in Bio.HPP^®^ as a dental restorative material stem from the balance between elasticity and rigidity, which resembles that of natural teeth [14]. The major advantage of Bio.HPP® endocrown restoration is a modulus of elasticity of (4 GPa) rendering it bone-like in its elasticity, and near to dentine, enabling it to function as a stress breaker to lessen the masticatory forces sent to the underlying tooth [15, 16]. This minimizes the vertical and horizontal stresses upon the remaining tooth structure and surrounding bone rather than other restorative material [16, 17].

Restorative material success relies on the quality and durability of the bond between resin cement and restoration. Lithium disilicate endocrowns offer superior retention and tensile bond strength compared to PEEK material [18]. LDS can be etched and bonded to dental structures. Hydrofluoric (HF) acid etching creates a porous surface while silane coupling agent provides chemical adhesion, forming siloxane bonds at the ceramic-resin interface [19]. Furthermore, surface treatments promote micromechanical interlocking of resin materials with adhesives. Sandblasting with Alumina particles improves bond strength. Visio.link is a good initial pretreatment for PEEK surfaces [20].

Fracture resistance of dental restorations is among the most crucial characteristics for the clinical success and durability of dental prosthetics. It relies on the material's ability to withstand internal flaws that might cause cracks propagation. These cracks might result in bulk fracture of the entire restoration or microscopic fractures of the restorative margins [21]. A restorative material's ability to resist fracture is influenced by a variety of parameters, including the angle at which the load is applied, and the magnitude of these forces. Teeth inside the oral cavity are subject to different force directions depending on their position and function. Posterior teeth, which are responsible for the tearing of food during mastication, are predominantly subject to vertical and oblique trajectory of forces than anterior teeth [22, 23]. This makes them more susceptible to fracture when they are significantly weakened by massive loss of coronal tooth substance following endodontic treatment [24].

The mode of failure of a tooth or a dental restoration is of paramount importance. Since fracture is one of the most commonly occurring clinical complications, the way in which a tooth or a restoration fails may vary, which in turn will directly affect the final decision of whether to restore or extract the tooth being treated. Failure analysis is a significant examination that helps in detecting the cause, origin, and direction of crack propagation [25]. On the other hand, ignorance of the nature, characteristics, and range of tooth structure fractures might be misinterpreted leading to inaccurate diagnostic conditions and unsuitable treatment. The type of treatment selected is greatly influenced by the extent, orientation, and location of cracks. Five categories of longitudinal fractures divided by the American Association of Endodontics have been developed to offer universal definitions that academics may utilize. According to that classification, Craze lines are limited to enamel. As opposed to fractured cusps, cracked and split teeth impact the enamel, dentin, and sometimes even the pulp or root, starting on the occlusal surface and extending apically [26].

The present research aimed to compare the fracture resistance of modified PEEK and lithium disilicate endocrown restorations on molar teeth and evaluate their failure mode. The null hypothesis of the current research was that there would be no significant variation in the fracture resistance and mode of failure between modified PEEK and lithium disilicate endocrown restorations.

## Materials and methods

Recently extracted 16 human mandibular second molars for periodontal purposes were gathered from the outpatient clinic. Teeth with complete root formation and approximate crown morphology with similar mesiodistal (9 ± 1 mm) and buccolingual (9 ± 1mm) dimensions were selected. Teeth included in the study were examined under magnification × 50 and ensured to have no evident caries, fracture lines, cracks, or any signs of internal or external root resorption. Teeth were cleaned using an ultrasonic scaler (UDS-A LED, Guilin Woodpecker Medical Instrument CO, Ltd., Guangxi, 541,004 P.R. China) to remove debris then disinfected in a solution of sodium hypochlorite (NaOCl) for 10 min and stored at 37ºC in distilled water until usage.

A total of 16 endocrown specimens were fabricated for use in the present research and allocated into two equivalent groups according to used materials (Table 1), Pressable modified polyether-ether-ketone (PEEK) BioHPP^®^ (*n* = 8) (bredent GmbH&Co.KG Weissenhorner Str.289250 Senden, Germany LOT NO 441913) and Pressable lithium disilicate glass–ceramic (LDS) IPS e.max^®^ (*n* = 8) Press (Ivoclar Vivadent AG FL-9494 Schaan\Liechtenstein LOT NO ZO2Y58). (Table 2).

**Table 1:** The material used in the study

Material	Manufacturer	Lot No
Pressable Lithium disilicate glass–ceramic ingots (IPS e.max^®^ Press)	Ivoclar Vivadent AG FL-9494, Schaan\Liechtenstein	Z02Y58
Pressable modified PEEK (BIOHPP) Pellet	bredent GmbH&Co.KG Weissenhorner Str.289250 Senden, Germany	441,913
VISIO.LINK	bredent GmbH&Co.KG Weissenhorner Str.289250 Senden, Germany	210,802
Porcelain etchant	BISCO, Inc.1100W. Irving Park Rd. Schaumburg, IL60193 USA 1–847-534–6000	2,200,000,408
Porcelain primer (Silane)	BISCO, Inc, Irving Park Rd. Schaumburg, IL USA	2,100,008,471
Bonding agent	All-Bond Universal®- BISCO, Inc., Irving Park Rd. Schaumburg, IL USA	2,200,005,591
Acid etchant	Select HV®ETCH, BISCO, Inc., Irving Park Rd. Schaumburg, IL USA	2,100,001,680
Dual-cure resin cement (BIS CEM)	BisCem®- BISCO, Inc. Irving Park Rd. Schaumburg, IL USA	2,200,000,549

**Table 2:** Chemical composition of IPS e.max^®^ Press and BIOHPP

Material	Chemical composition
Pressable Lithium disilicate glass–ceramic ingots (IPS e.max^®^ Press)	SiO_2_, LiO_2_, K_2_O, P_2_O_5_, MgO, ZnO, and other coloring oxides
Pressable modified PEEK (BIOHPP) Pellet	Polyether ether ketone with 20% (by weight) inorganic content (ceramic filler)

Each tooth was mounted in a self-cured acrylic resin (Acrostone, Cold cure, ISO13485, England) block after the application of a uniform layer (0.2 mm) of molten dental wax (Tauchwachs, Dipping wax, BEGO, Lot 740,371, Germany) covering the embedded portion of the roots 2 mm below the cemento-enamel junction down to the root apex. Following the complete setting of the self-cure acrylic resin blocks, wax was eliminated followed by injection of light body silicone (Ghenesyl, ISO 4823-TIPO 3, LASCOD Spa, Italy) material around the roots and tooth was repositioned in its mold, this step was performed to simulate periodontal ligament membrane.

Tooth prepared by a single experienced clinician with the aid of a dental surveyor (BEGO, PARASKOP M, Model No: 26060, S/N: 288 799, 100–120/200-240V, 50/60HZ, D-28359 Bremen). Milling surveyor machine was used in this study to give a more standardized preparation with the elimination of the human errors and mistakes as much as possible. A low-speed straight hand piece (Strong Traus AT-II, Saeshin Precision Co., Korea) with adaptor (Intensive SA, H 2003, Montagnoia, Switzerland) was fixed on the dental surveyor. Each specimen was supported by a base of stone (Elite dental stone, Zhermach, LOT 0000379788, Italy) and a periodontal probe was used to standardize all measures. Using a blue permanent thin marker pen, the mesial and distal sides were marked at 3 mm occlusal to the CEJ using a graduated periodontal probe to reduce occluso-gingival height in a standardized manner.

Occlusal preparation was initiated using abrasive disks to make the horizontal cut. The internal cavity of the coronal portion surface was done using a round bur to access the pulp chamber for endodontic treatment, followed by a standard butt-joint margin with a minimal 2 mm axial wall thickness. The internal axial walls of the cavity were prepared using a standardized tapered stone (#534, code 235AC, Lot no. FG.09.21.06481, Switzerland) to remove any existing undercut within the pulp chamber, producing a cavity with 8–10º coronal divergence, keeping 2 mm of the tooth structure and 4 mm of intra-coronal cavity from occlusal platform of the butt joint to the floor of the pulp chamber.

The working length of each tooth was measured with #10 K-files (MANI, Lot R21K041000, Italy), with the aid of a periapical radiograph. The root canal instrumentation (cleaning and shaping) was performed with machine-driven (COXO, C-Smart-1, MCS, CE0197, China) rotary files (ROGIN DENTAL, NITI ROTARY FILES, FDA CE0197, Lot R-P 104224, China), following the sequence Orifice Opener, F1, F2, F3. The canal was irrigated with 5.25% NaOCl and EDTA during the preparation process. The root canals were then dried with absorbent paper tips. Root canals were obturated with gutta-percha points that were coated with a resin-based sealer (ADSEAL^®^). Next, the floor of the pulp chamber was covered with a thin layer of a dental adhesive bonding agent and Flowable resin composite and fully cured. Each specimen was placed in a small, coded jar filled with distilled water and stored in an incubator at 37°C to avoid its dryness.

Fully dental upper and lower gypsum casts were laser scanned to obtain a 3D digital image stereolithography (STL file) for both arches. Computer software (Realguide^®^ v5.0 3diemme, Italy) was then used to modify the digital 3D model, removing the mandibular left second molar from its corresponding arch model, creating a tube-like space to be occupied by the tooth and its corresponding acrylic mold in a later stage. Physical models of the digitally modified casts were obtained by 3D printing (Creality CR-10 printer, China). Each specimen with its acrylic mold was placed into the tube-like space in the mandibular second molar region. Both upper and lower mandibular casts were sprayed for a scan (Occlutec, No. 19350000, Renfert, England), and then placed in an intercuspal position. The maxillary and mandibular 3D printed casts with the tooth in place were scanned using a bench top extra oral scanner (Dental Lab Scanner FREEDOM (DOF) company extraoral scanner, Korea) to obtain a digital model of the prepared tooth within the model.

Endocrown wax patterns were digitally designed using dental CAD software (ExoCAD version 2.3, Germany) which ensured a uniform occlusal thickness of 2 mm. Wax patterns were milled using a CAM dental milling machine (Roland Milling machine, DWX-510, Japan) and checked to fit the corresponding tooth. The pressing procedure for BioHPP^®^ and IPS e.max^®^ was completed according to the manufacturer’s recommendations for each material. All endocrown samples underwent a 10-min ultrasonic cleaning in distilled water and were left to air dry. The intaglio surface of each IPS e.max^®^ press endocrown was etched using 9.5% hydrofluoric acid porcelain etching gel (BISCO Inc, USA, Lot No. 22000008544) for 20 s, and then rinsed off using air–water spray for 60 s. While intaglio surface of each BioHPP^®^ endocrown was air abraded with Aluminum oxide particle of 110 µm at air pressure of 2 bar for 10 s at a standardized 10 mm distance from the tip of sandblasting device to the specimen surface.

Before primer application, all endocrown restorations were cleaned in an ultrasonic bath for 4 min in distilled water, followed by drying with oil-free compressed air. A porcelain primer which contains silane as a coupling agent (BISCO Inc, USA, Lot No. 2100008551) was applied to the etched intaglio of IPS e.max^®^ specimens. A thin layer of Visio.link^®^ primer (bredent GmbH & Co. KG, Germany, Lot No. 210802) was applied to the sandblasted surface of BioHPP^®^ specimens then photo-polymerized using the manufacturer’s recommended light curing device (Bredent GmbH & Co. KG, Germany).

The enamel of every prepared tooth was selectively etched using 35% phosphoric acid gel for 15 s (Select HV^®^ETCH, BISCO Inc., USA, Lot No. 2100001026), and then rinsed off using air–water spray for 30 s, followed by gently air-dried. A dental universal adhesive bonding agent (All-Bond Universal^®^- BISCO Inc., USA, Lot No. 2200005591) was applied to the prepared tooth surfaces, rubbed by micro-brush then air-thinned and light cured for 20 s. Dual-cure resin cement (BisCem^®^, BISCO Inc., USA, Lot No. 2200000549) was used to cement the endocrowns. Each endocrown was seated onto its corresponding preparation under a 5 KG static load applied in a straight line using a loading mechanism, excess cement was tack cured for 2 s, then a sharp explorer was used to remove extra cement, and final light curing for 20 s for every surface was done.

After the cementation process, the endocrowns were exposed to thermal cycling and were subjected to a chewing simulator. Thermocycler THE 1100 (SD Mechatronik GMBH in Miesbacher, Feldkirchen-Westerham, Germany) was used to perform thermal aging. The water level in the system was checked daily and adjusted, if necessary, without affecting the water bath temperature. The water bath temperature ranged 5–55°C, with bath/dwell cycles of 10-60s. After undergoing thermocycling, the specimens were subjected to mechanical aging in a multimodal chewing simulator (Robota, Model ACH-09075DC-T, ADTECH Technology Co., LTD., Germany). A stainless-steel antagonist stylus was employed to transfer a 10 kg weight (98 *N* of chewing force) vertically directed toward the central fossa of the occlusal surface. The test parameters were 1.6 Hz cycle frequency, and 2.4 N.m. torque. Each specimen underwent chewing simulation for 118,000 cycles, which represents one year of clinical performance [27].

The fracture of the prostheses was caused by the fracture test. The Bluehill Lite Software (Bluehill Lite Software, Instron^®^, USA) was used to carry out fracture resistance tests. The fracture of all specimens was caused by the fracture test. After every specimen was individually mounted on a computer-controlled materials testing machine (Instron Industrial Products, Norwood, MA, USA) via a load cell of 5 kN, data were collected utilizing computer software. By tightening screws, samples were fastened to the lowest stable chamber of the testing machine. To minimize the transmission of local force peaks while achieving an equal distribution of stress, the fracture test was conducted via a compressive manner of load-delivered occlusal utilizing a steel rod fixed to the top moveable chamber of the testing machine and moving at a crosshead speed of 1 mm/min. The steel rod with a spherical tip (8.6 mm in diameter), that used as the force applicator, was vertically directed toward the central fossa of the occlusal surface of endocrown. The failure mode was evaluated by inspecting fractured surfaces using a stereomicroscope and further examined by scanning electron microscope (SEM).

Statistical analysis was performed with SPSS (Statistical Package for Social Science, version 20, IBM, USA), Graph Pad Prism (Graph Pad Technologies, USA), and Microsoft Excel. All quantitative data was presented as mean and standard deviation, while all qualitative data were presented as frequency and percentages. All data were presented in tables and graphically represented in figures. Results of fracture resistance of tested groups were analyzed using independent t-test. Meanwhile, mode of failure was statistically analyzed using the Chi Square test. Shapiro–Wilk and Kolmogorov–Smirnov tests for normality revealed a statistically non-significant difference for each group (P > 0.05) indicating that data originated from parametric data was in normal distribution in both groups.

## Results

Descriptive numerical data of fracture resistance were expressed as minimum, maximum, median, mean, standard deviation, standard error, and confidence interval at 95% of both groups and presented in Table 3.

**Table 3:** Descriptive statistics for fracture resistance of both groups in Newton

Group	Min	Max	Med	Mean	SD	SE	95% CI	
							Lower arm	Upper arm
**PEEK**	2527.19	3200.14	2744.92	2762.96	216.15	76.42	2582.25	2943.67
**LDS**	1749.88	2501.85	2195.75	2175.91	267.72	94.65	1952.08	2399.73

*Min* minimum, *Max* maximum, *Med* median, *SD* standard deviation, *SE* standard error, *CI* confidence interval

Mean fracture resistance of both groups revealed that the mean load required for fracture of modified PEEK endocrowns was (2762.96 ± 216.15 *N*), while for LDS endocrowns it was (2175.91 ± 267.72 *N*). An independent t-test statistical analysis showed that both groups' fracture resistance differed statistically significantly, as presented in Table 4.

**Table 4:** Mean and standard deviation of load at fracture in both groups and comparison using independent test

Group	Mean	SD	Difference (Independent t-test)				
			MD	SED	95% CI		***P*** **value**
					Lower arm	Upper arm	
**PEEK**	2762.96	216.15	587.06	121.65	326.14	847.97	0.0001*
**LDS**	2175.91	267.72					

*MD *mean difference,* SED *standard error difference,* CI *confidence interval

^*^Significant difference as* P* *< 0.05*

**Fig. 1:**
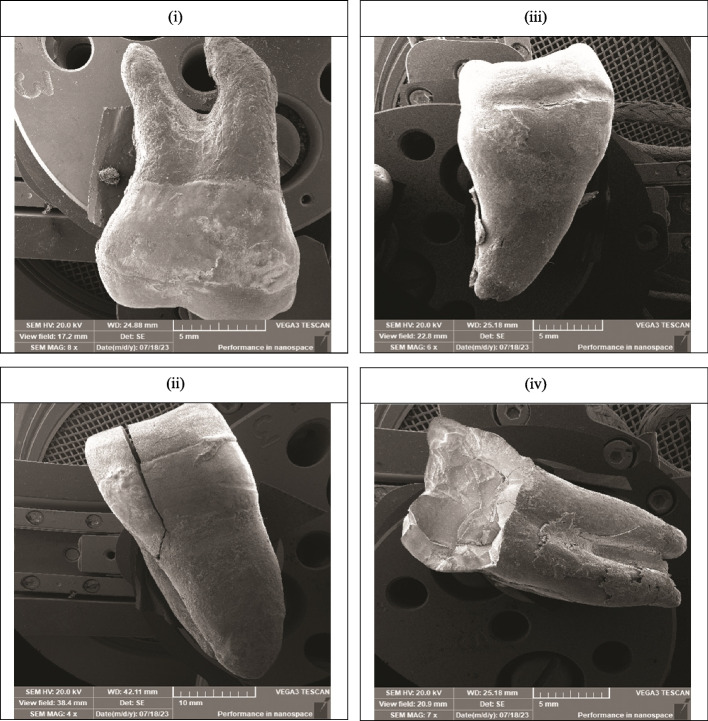
Scanning Electron Microscope images (SEM) showing different failure modes. i: PEEK deformation with restorable tooth, ii: PEEK deformation with non-restorable tooth, iii: LDS deformation with restorable tooth, iv: LDS deformation with non-restorable tooth

**Fig. 2:**
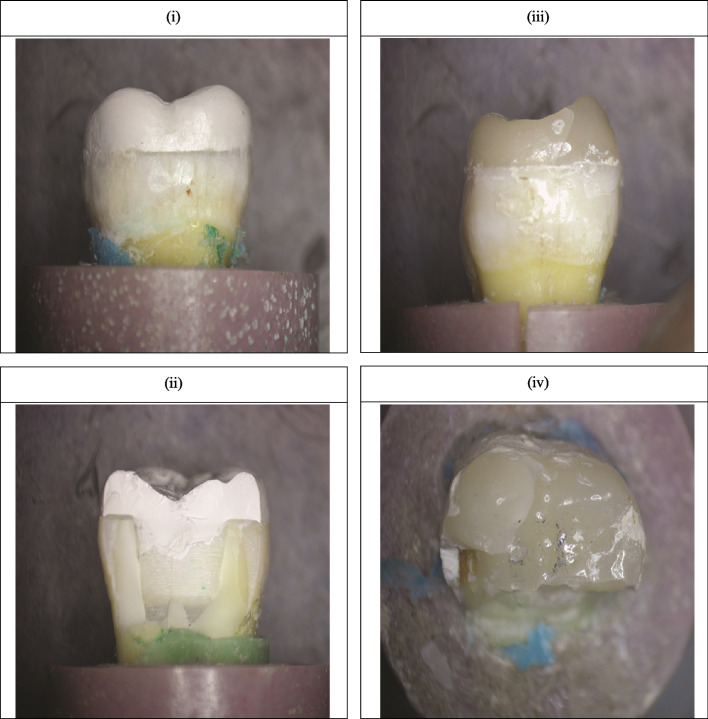
Stereomicroscope images showing different failure modes. i: PEEK deformation with restorable tooth, ii: PEEK deformation with non-restorable tooth, iii: LDS deformation with restorable tooth, iv: LDS deformation with non-restorable tooth

Fractured parts of each specimen were inspected using SEM (Fig. 1) and Stereomicroscope (Fig. 2) in both groups and the mode of failure was assessed according to the parameters set by the American Association of Dental Society classified into five classes of failure is presented in Table 5 as frequency, percentage, and statistical analysis of the results of each group.

**Table 5:** Frequency and percentage of different failure modes regarding tooth structure and restoration in both groups

Affected Part	Mode of failure	PEEK		LDS		Pearson Chi-Square Tests		
		*N*	%	*N*	%	Chi-square	Df	*P* value
**Tooth structure**	Craze line	0	0	0	0	6.000	5	0.30
	Fractured cusp	2	25.0	4	50.0			
	Cracked tooth	1	12.5	1	12.5			
	Split tooth	1	12.5	2	25.0			
	Vertical root fracture	1	12.5	0	0.0			
**Restoration**	Restoration deformation with unaffected tooth	3	37.5	0	0.0			
	Restoration fracture with unaffected tooth	0	0.0	1	12.5			
	Restoration debonding	0	0.0	0	0.0			

*N *Count,* % *Percentage,* P *probability level which is significant at* P* *< 0.05*

**Fig. 3:**
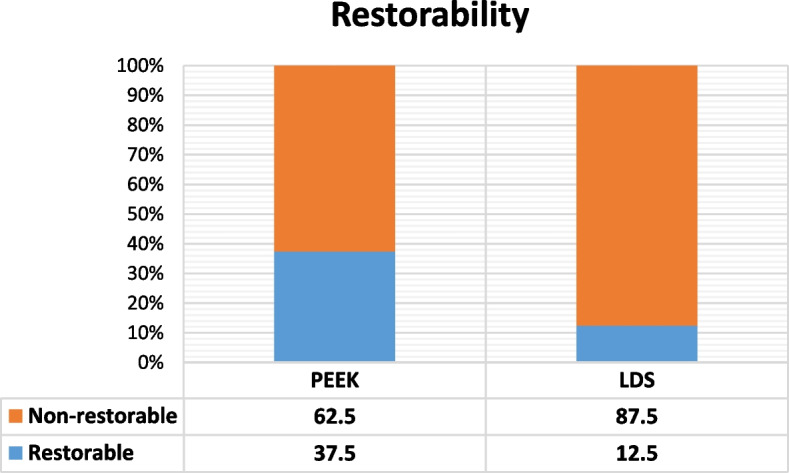
A stacked column chart showing the restorability percentage in both groups

Chi-square test statistical analysis utilized to contrast failure mode in both groups revealed a non-significant statistical difference between PEEK and LDS. The frequency of restorable/non-restorable tooth fractures and percentages are graphically represented in (Fig. 3).

## Discussion

The preferred endocrown material is crucial because it affects how biomechanical stress is distributed, which in turn affects how long endodontically treated teeth last [28]. LDS is considered one of the best restorative options for these teeth; because of its superior optical qualities, strong fracture resistance, and adhesive qualities [28, 29].

Today, IPS e.max^®^ restoration is available as pressed (IPS e.max^®^ press) and IPS e.max^®^ CAD. The heat-pressed IPS e.max^®^ Press shows larger crystals in length compared to that of machinable IPS e.max CAD-CAM [10]. Owing to these minute variations in the size and structure of the crystal, the flexural strength of IPS e.max^®^ Press is 400 MPa more than that of IPS e.max^®^ CAD, which is 360 MPa. Heat-pressed LDC appeared to be less susceptible to aging than milled LDC. When looking at strength, IPS e.max^®^ press is 11% stronger than IPS e.max^®^ CAD [10, 30].

PEEK is an alternate restorative material that can be used, especially after modification by Ceramic filler (BioHPP^®^), which produces constant homogeneity and improve the strength, and abrasion properties [14]. The main benefit of this modified PEEK material is its low modulus of elasticity, which makes it bone-like in its elasticity. This property enables it to function as a stress breaker, therefore reducing the stresses applied to the tooth root and the restoration [15, 16].

BioHPP^®^ pressed pellets were used as it showed higher mechanical properties and stability than those pressed granules [31]. Industrial pre-pressing of pellets increases the stability, reliability, and flexural strength of the material of PEEK restorations [31, 32].

Due to their bonding properties, heat conductivity, modulus of elasticity, and strength, which are more similar to clinical conditions, human teeth were utilized in the present research rather than those from cattle, metal, or acrylic materials [8].

Molar teeth were selected for endocrowns restoration; as the reduced dental structure in the premolar reduces adhesive surface area, while longer lever arms in premolars (due to their larger height than width) increase the risk of adhesive failure and dislocation in endocrown restoration [33]. Also, a Butt joint preparation design was selected; because it showed a higher fracture resistance at a much greater load when compared to the ferrule preparation design of an endocrown [34].

The difference in thickness of restoration affects fracture resistance. Used upper and lower 3D printing casts and wax pattern fabricated using CAD-CAM technique; were to ensure standardization of thickness of endocrown restoration [35, 36]. Using acrylic resin with the addition-silicon light body impression material in a simulation of periodontal ligament may provide a more accurate in vitro reproduction of the oral condition. The periodontal ligament is an essential component that helps in stress distribution produced by the load performed over the tooth. It is shown that periodontal ligament simulation has a bigger impact on the fracture mode than the fracture load values [37].

Physical surface treatments used by sandblasting with 110 μm aluminum oxide proved to increase surface roughness, creating fresh surface layer via cleaning the surface of the substance of organic contaminants, promoting micromechanical interlocking of polymer-based dental materials with dental adhesives; as the bond strength is increased by irregularities in the bonding region [20]. Visio.link^®^ PEEK bond was selected for adhesive systems; the chemical composition is methyl methacrylate (MMA) and pentaerythritol triacrylate (PETIA). Visio.link^®^ gave PEEK restorations the highest bond strength scores due to PETIA's great ability to alter the PEEK surface [38]. A stronger bonding strength will be achieved with IPS e.max Press surface treatment utilizing 9% HF for 20 s as compared to 20 s with 5% HF acid [19]. HF acid surface treatment results in insoluble fluorosilicate salts, that may reduce the molecular interaction within the ceramic and the resin cement [39]. The best method used to remove the precipitates of fluorine deposits is placing the specimen in an ultrasonic bath with distilled water [40]. Such processes enhanced the bonding strength of resin cement and lithium disilicate [40, 41].

Silane coupling agents are used to serve as intermediaries that, by their dual reactivity, promote adhesion between various inorganic and organic components. By creating siloxane linkages at the resin-ceramic contact, silanes are useful for strengthening the bond [18, 42]. A thermomechanical cycle chewing machine is a tool that mimics the mouth and the jaw's masticatory function. Endocrowns specimens were thermomechanical cycled for 118,000. Cycles are required whenever applying a larger load of 100 N in order to replicate one clinical year [27]. Biomimetic dentistry revolutionized the repair and replacement of diseased dental tissues, with two main groups: stress-reducing and bond-maximizing protocols. The material used should suit the tooth's function and modulus of elasticity [43, 44]. The elastic modulus of dentin is 14.7 GPa, LDS is 95 ± 5 GPa, while PEEK is 3–4 GPa [45].

The null hypothesis of the current research that there will be no significant variation in the fracture resistance and mode of failure between modified PEEK and lithium disilicate endocrown restorations was partially rejected. Considering the present research's findings, modified PEEK had a statistically significant higher fracture resistance than LDS endocrown restorations. This can be explained by the main PEEK characteristic, which is low elastic modulus, mimics that of human bone, reducing the maximum values of masticatory forces, both laterally and vertically, comparatively with ceramic material [46, 47]. If a restorative material which has a higher elastic modulus compared to dentin is chosen, the more rigid restoration may cause wedging action on the entire tooth-restorative system. However, using a substance with an elastic modulus that is comparable to dentin enables the restoration to exhibit biomechanical behavior similar to that of tooth structure, allowing them to act as one unit and act as shock absorbable to high occlusal load [45, 46, 48]. This similarity in the elastic modulus between modified PEEK and dental tissues allows it to serve as a stress breaker and, as a result, lessen the pressures that are transmitted from the restoration to the tooth root [15].

One possible substitute material for restoration is PEEK, especially after modification by ceramic fillers which produce constant homogeneity and improve the strength and abrasion properties [49].

According to the findings of the present research, the lowest load causing failure of specimen in BioHPP was (2527.19 *N*) and maximum load was (3200 *N*). Whereas, for LDS endocrowns, the minimum load was (1749.88 *N*) and the maximum was (2501.85 *N*). This shows that the minimum load required to fracture modified PEEK specimens was more than the maximum load applied for LDS endocrowns. This higher stress-bearing capacity of modified PEEK may help reduce stresses transferred from the restoration to the underlying tooth structure complex.

In clinical practice, both BioHPP and lithium disilicate endocrowns may be utilized safely regarding resistance to fracture, as the two possess minimum load causing failure (2527.19 *N* and 1749.88 N, respectively), which exceeded the physiologic masticatory load in the posterior region (300–500) *N* [50, 51]. BioHPP is a recommended treatment for parafunctional patients with bruxism due to its ability to withstand large biting forces, maintain tooth structure without fracture, and demonstrate high resistance to abrasion for antagonists, especially when esthetic concerns are not a major concern [52]. LDS, a popular option for endodontically treated molar teeth restoration, offers etchable and adhesively bondable endocrowns. It is available as pressable ingots or CAD-CAM blocks, pressable LDS IPS e.max^®^ Press has higher flexural strength, less aging vulnerability, superior aesthetics, and superior bonding to dental structures [9, 10].

The outcomes of the current research were consistent with many previous studies [45, 47, 53, 54], they concluded that peek endocrowns displayed significantly greater fracture resistance over e.max even up to eight times. The explanation of their results was owing to the modulus of elasticity and the inherent property of integrated crack prevention of peek material.

Nevertheless, other studies [55, 56] showed contradictory results to our study. However, they used premolar teeth restored by endocrowns and used different aging method (5,000 thermocycles) equivalent to 6 months without mechanical loading, also the failure mode was within composite veneering material of bi-layered peek specimens [55].

According to the outcomes of the present research, the second part of the null hypothesis that there will be no significant variation in mode of failure between modified PEEK and LDS endocrowns was accepted. Statistical analysis of the number of specimens with catastrophic tooth fractures in each two groups revealed a statistically non-significant difference.

The study reveals that modified PEEK endocrowns have a higher frequency and percentage of favorable tooth fractures compared to LDS endocrowns. After endocrown specimens’ fracture, 37.5% of restored teeth showed favorable restorable fractures, compared to 12.5% in teeth restored with LDS endocrowns. This favorable failure mode is attributed to modified PEEK's superior ability to absorb energy causing fractures through elastic deformation.

With regards to the clinically repairable mode of failure, the present research's findings were in agreement with a study by Abd El Rahman et al. [55] who concluded that PEEK recorded a higher percentage of restorable tooth failures that avoided the possibility of catastrophic tooth fracture compared LDS endocrowns. Furthermore, Leon et al. [57] who studied fracture resistance of LDS and composite-veneered bi-layered PEEK endocrowns reported predominant sub-bony tooth fracture 2 mm below the cemento-enamel junction in teeth restored with LDS endocrowns, which is considered as catastrophic unfavorable failure. They also reported that every fracture in the PEEK group occurred inside the veneering composite material, rendering failures in this group 100% favorable and repairable with no catastrophic tooth fracture.

Disagreement with the present research's findings is noted in a study conducted by Elkady et al. [47] who reported similar unfavorable failures in both lithium disilicate and air-abraded PEEK endocrowns in their research. Additionally, Aher et al. [56] showed that both LDS and PEEK materials exhibited mainly catastrophic failures with no significant differences.

Even though standardization and clinical relevance of the steps of this in-vitro study were meticulously followed and assured, anticipation of the clinical performance of dental treatments can vary in clinical practice. Hence, interpretation of results of laboratory tests and drawing clinical recommendations must be taken cautiously. For this reason, repetition of the laboratory steps clinically and intraorally is necessary to obtain actual results for both materials. In the present research, specimens were vertically loaded. Further studies are necessary to explain the effect of lateral loading on the fracture resistance especially on PEEK that has adhesive performance less than lithium disilicate.

## Conclusion

Under the limitations of the present study, the following could be concluded:

1. Modified PEEK (BIOHPP^®^) endocrowns have a greater fracture resistance over lithium disilicate (LDS) endocrown restorations.
2. Both modified PEEK and lithium disilicate endocrowns may be utilized safely regarding fracture resistance because both have values higher than what is needed for physiology.
3. The likeliness of catastrophic fracture of endodontically treated molars restored with BIOHPP^®^ is less than teeth restored with LDS endocrowns.

## Data Availability

The dataset used and analyzed data are available from the corresponding author upon reasonable request.
